# Supplementary material for: The Willingness to Pay for a Hypothetical Vaccine for the Coronavirus Disease 2019 (COVID-19)
Source: Int J Environ Res Public Health. 2021 Nov 26;18(23):12450. doi: 10.3390/ijerph182312450 (PMC8657379; doi:10.3390/ijerph182312450)
Supplement: Supplementary file 1 [file ijerph-18-12450-s001.zip › ijerph-1445521-supplementary.pdf]

Supplemental Online Content

The willingness to pay for a hypothetical vaccine
for the coronavirus disease 2019 (COVID-19)

Yoshiro Tsutsui, Shosh Shahrabani, Eiji Yamamura,
Ryohei Hayashi, Youki Kohsaka, Fumio Ohtake

Figure S1. Distribution of the answers of VACCINE for each wave

Figure S2. Distribution of the answers of SEVERITY for each wave

Figure S3. Distribution of the answers of probability of infection (PROB) for each wave

Figure S4. Distribution of risk attitude of each wave

Figure S5. Magnitude of the variables at each wave

Figure S6. Sensitivity of VACCINE on each variable at each wave

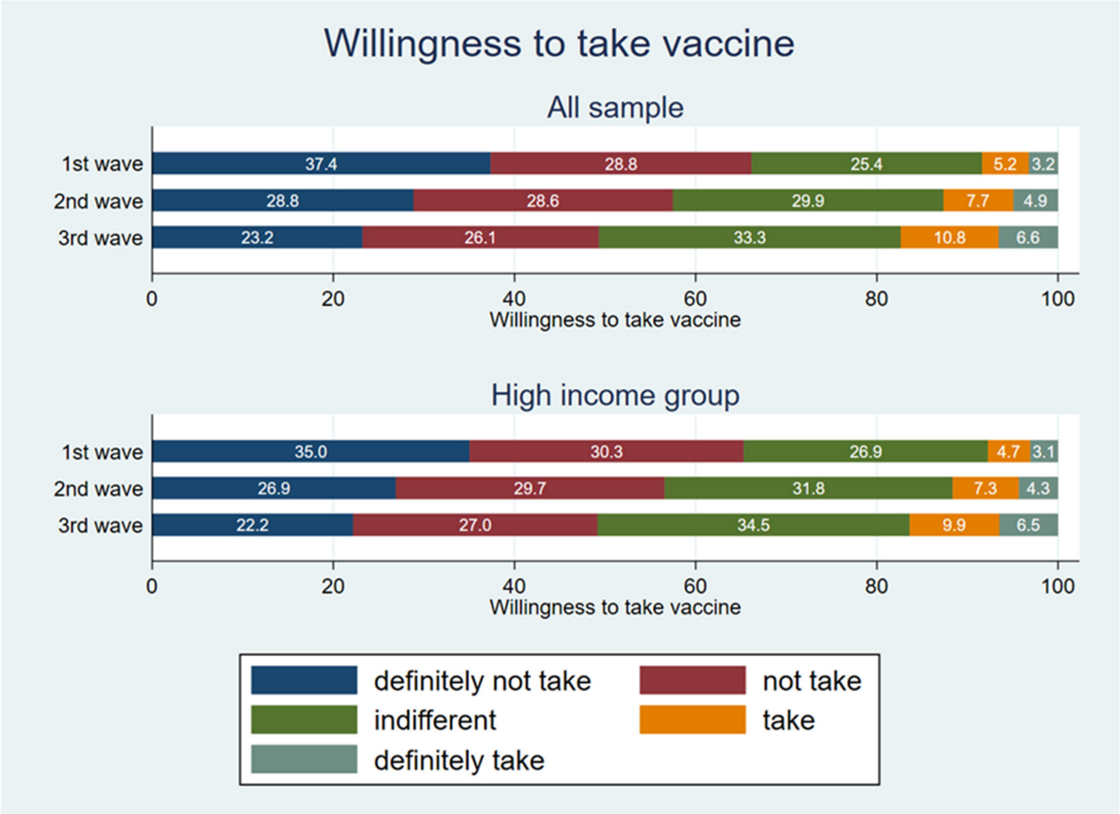

Figure S1. Distribution of the answers of VACCINE for each wave

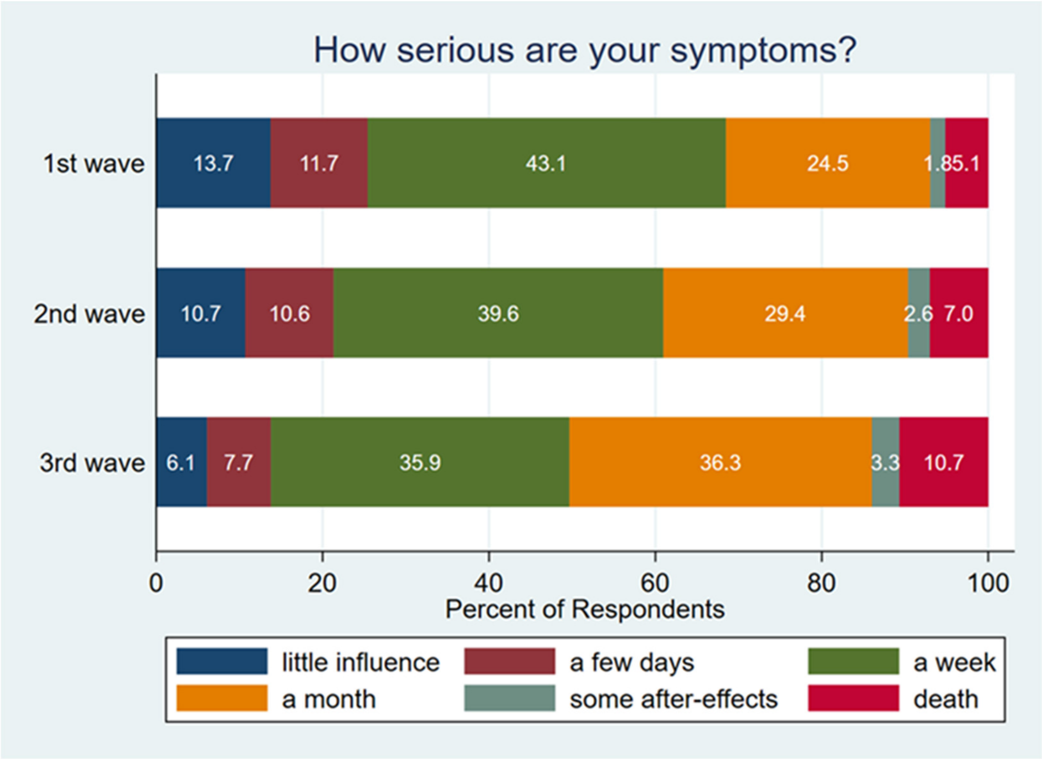

Figure S2. Distribution of the answers of SEVERITY for each wave

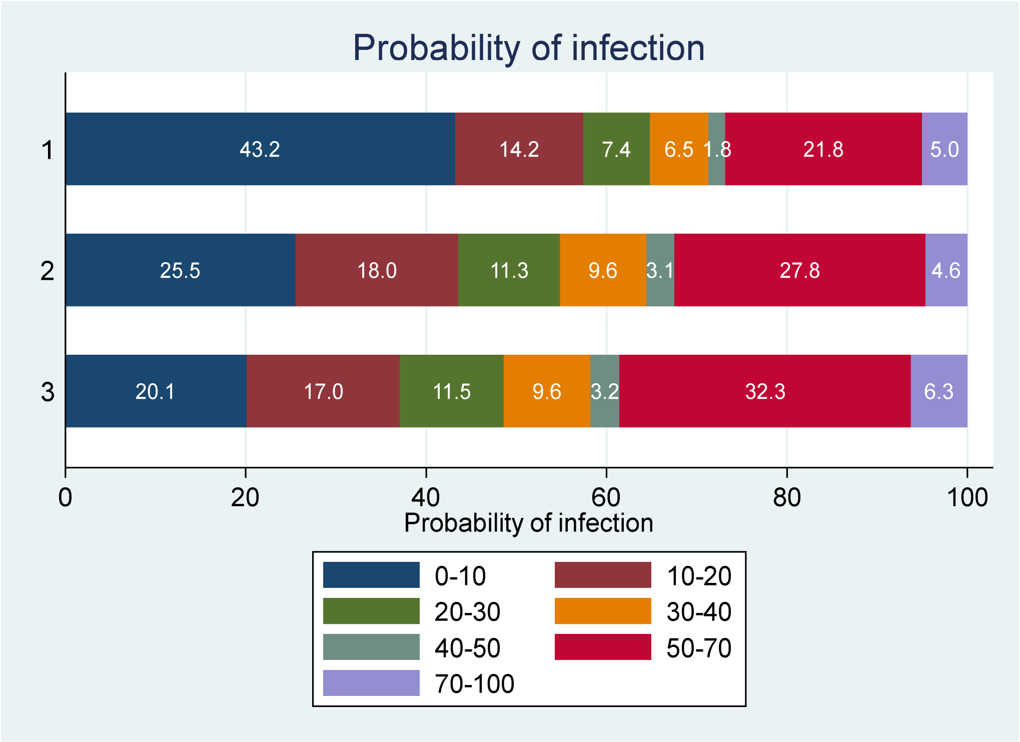

Figure S3. Distribution of the answers of probability of infection (PROB) for each wave

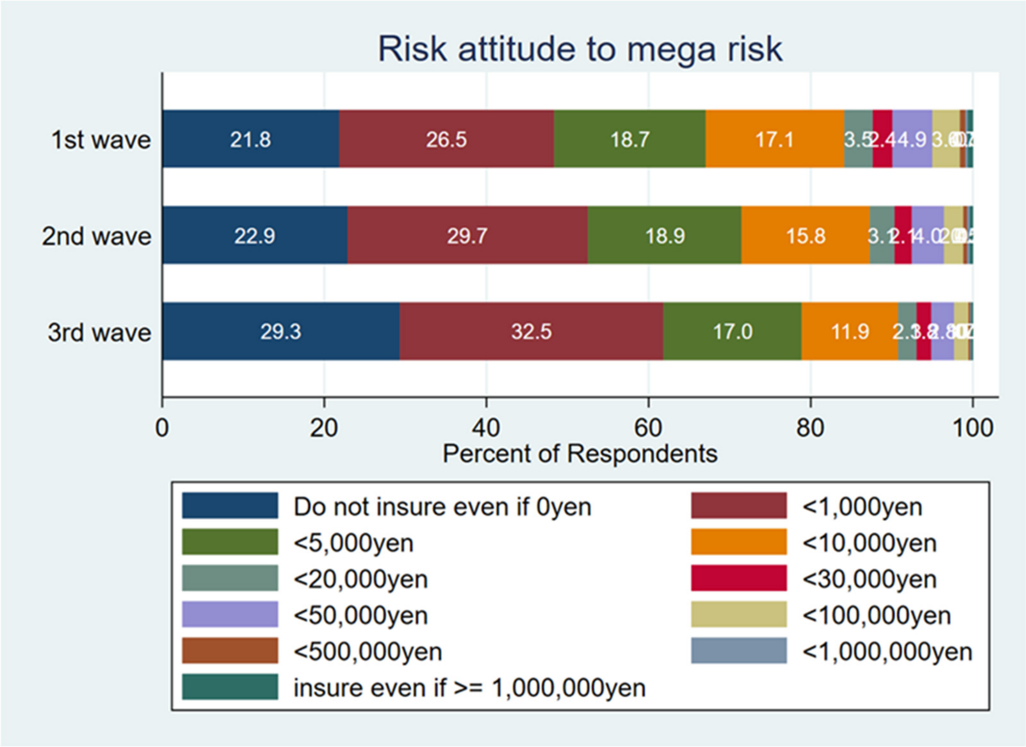

Figure S4. Distribution of risk attitude of each wave

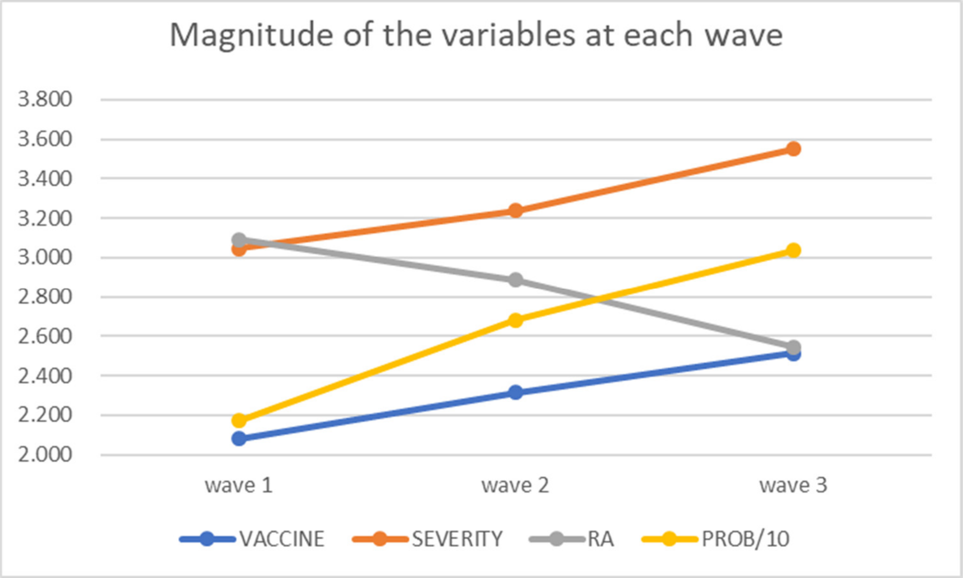

Figure S5. Magnitude of the variables at each wave

Note: Mean of PROB is divide by 10.

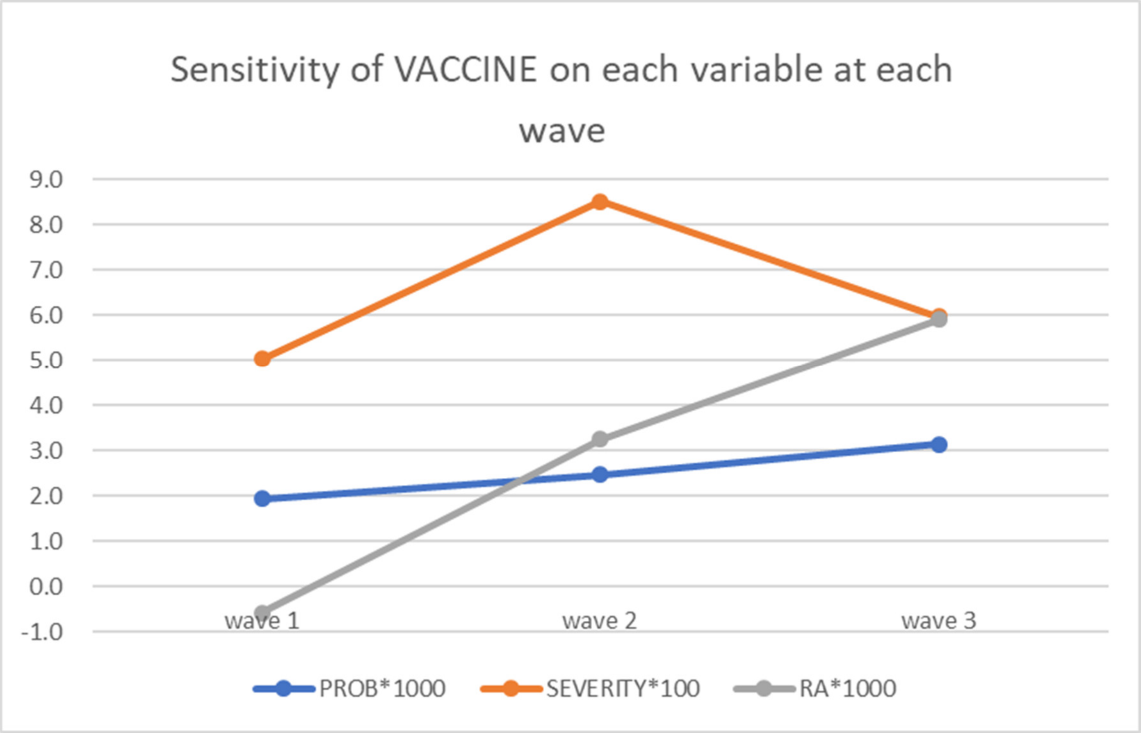

Figure S6. Sensitivity of VACCINE on each variable at each wave

Note: The coefficients of PROB and RA are multiplied with 1000 and that of SEVERITY with 100.
